# Exploring Mitogenomes Diversity of *Fusarium musae* from Banana Fruits and Human Patients

**DOI:** 10.3390/microorganisms10061115

**Published:** 2022-05-28

**Authors:** Luca Degradi, Valeria Tava, Anna Prigitano, Maria Carmela Esposto, Anna Maria Tortorano, Marco Saracchi, Andrea Kunova, Paolo Cortesi, Matias Pasquali

**Affiliations:** 1 Department of Food, Environmental and Nutritional Science (DeFENS), University of Milan, Via Celoria 2, 20133 Milan, Italy; luca.degradi@unimi.it (L.D.); valeria.tava@unimi.it (V.T.); marco.saracchi@unimi.it (M.S.); andrea.kunova@unimi.it (A.K.); paolo.cortesi@unimi.it (P.C.); 2 Department of Biomedical Sciences for Health, University of Milan, Via Pascal 36, 20133 Milan, Italy; anna.prigitano@unimi.it (A.P.); maria.esposto@unimi.it (M.C.E.); annamaria.tortorano@unimi.it (A.M.T.)

**Keywords:** cross-kingdom pathogen, *F. fujikuroi* species complex, mitochondrial diversity

## Abstract

*Fusarium musae* has recently been described as a cross-kingdom pathogen causing post-harvest disease in bananas and systemic and superficial infection in humans. The taxonomic identity of fungal cross-kingdom pathogens is essential for confirming the identification of the species on distant infected hosts. Understanding the level of variability within the species is essential to decipher the population homogeneity infecting human and plant hosts. In order to verify that *F. musae* strains isolated from fruits and patients are part of a common population and to estimate their overall diversity, we assembled, annotated and explored the diversity of the mitogenomes of 18 *F. musae* strains obtained from banana fruits and human patients. The mitogenomes showed a high level of similarity among strains with different hosts’ origins, with sizes ranging from 56,493 to 59,256 bp. All contained 27 tRNA genes and 14 protein-coding genes, rps3 protein, and small and large ribosomal subunits (rns and rnl). Variations in the number of endonucleases were detected. A comparison of mitochondrial endonucleases distribution with a diverse set of *Fusarium* mitogenomes allowed us to specifically discriminate *F. musae* from its sister species *F. verticillioides* and the other *Fusarium* species. Despite the diversity in *F. musae* mitochondria, strains from bananas and strains from human patients group together, indirectly confirming *F. musae* as a cross-kingdom pathogen.

## 1. Introduction

Different fungal species are able to cross hosts, causing diseases not only in plants but also in humans and animals. In general, cross-kingdom fungi are weak pathogens for both plants and humans; they can also be asymptomatic in plants but can have clinical significance, especially in people with impaired immunity or those who have sustained penetrating trauma [1]. The role of food and agriculture in the transmissibility of cross-kingdom pathogens deserves to be studied accurately. 

*Fusarium musae* VanHove is a pathogenic species belonging to the *F. fujikuroi* species complex [2]. It causes crown rot in bananas, a post-harvest disease [3,4], and it also causes keratitis and skin infections as well as systemic infections in immunocompromised patients [5,6]. 

Based on multilocus sequence typing, *F. musae* can be distinguished from its sister species *F. verticillioides* [2]. The two species show a diverse susceptibility to azoles, with *F. musae* having a higher tolerance to some fungicides compared to *F. verticillioides* [7].

*F. musae* has been identified on banana fruits in Central American regions, in the Philippines and Canary Islands and in European and Japanese markets of banana fruits. In patients, it has been identified in the US and Europe [5,8]. Understanding the level of diversity of strains infecting humans and banana fruits may help address the question about the transmissibility of *F. musae* in different hosts [9].

Mitochondrial genomes evolve independently of and faster than the nuclear genome [10]. Mitogenomes have also been proposed as a useful tool for diagnostic purposes in *Fusarium* species [11]. The concept of using mitogenome diversity for the identification of species mainly derives from their higher DNA copy number compared with nuclear DNA, and hence the high recovery and amplification success in eukaryotes where insufficient phylogenetic signals have accumulated in nuclear genes [12].

Fungal mitochondrial genomes are typically small, circular and double-stranded DNA molecules, with a typical set of mitochondrial genes with identical gene order. Extensive mitochondrial genome comparisons within the fungal kingdom have shown that any gene has a mitochondrial localization in all fungal species [13], suggesting that the use of mitochondrial gene diversity cannot be applied as a universal marker for fungi. Nonetheless, the analysis of mitochondrial diversity can be successfully applied to differentiate species within orders [14] or species [15,16]. For example, a unique feature apparently common in all *Fusarium* species is the presence of a large open reading frame with an unknown function (LV-uORF) firstly described in mitogenomes of *F. graminearum*, *F. verticillioides* and *F. solani* [17] and probably acquired prior to the divergence of *Fusarium* species.

In addition, fungal mitogenomes harbour a variable number of mobile genetic elements (MGEs) such as intron-associated homing endonucleases (HEGs), which have invaded mitogenomes throughout their evolution. Most of the MGEs insertion sites are highly conserved [18] and occur in mitochondrial protein-coding genes but can display remarkably different MGE densities. The same MGEs can be irregularly distributed in evolutionarily distant species and mosaicism in MGE patterns can be found between different populations or even strains of the same species, driving large genome size differences among them. Exploring MGE distribution can help discriminate species and subgroups, tracking the spread of fungal populations [19].

We, therefore, explored the mitogenomes of a collection of 18 *F. musae* strains in order to describe their mitochondrial diversity. The other objective of this study was to discover whether the pattern of MGE diversity might facilitate the identification of *F. musae* species within the *Fusarium* genus. Moreover, we explored whether *F. musae* from bananas and human patients shared the same mitochondrial sequence in order to see if genetic subgroups associated with the host origin could be identified. 

## 2. Materials and Methods

### 2.1. DNA Extraction and Sequencing

DNA used for sequencing was obtained from fresh mycelia of 16 strains according to a modified CTAB method [20], followed by Genomic tips column purification (Qiagen, Germantown, MD, USA). Sequencing was carried out using Illumina Hiseq 2000 (151 bp x2) by Novogene (Cambridge, UK). Mitogenomes of NRRL25059 [21] and F31 [22] strains were obtained from the NCBI database.

### 2.2. Other Fungal Mitochondrial Genomes

Mitogenomes from fungal strains belonging to different *Fusarium* species (see supplementary Table S1) were retrieved from NCBI and partially annotated if the annotation was missing (protein genes) using MFannot online software (University of Montreal, Montreal, QC, Canada), (https://megasun.bch.umontreal.ca/cgi-bin/mfannot/mfannotInterface.pl (accessed on 4 March 2022)).

### 2.3. Assembly and Annotation of Mitogenome

Assembly for all *F. musae* mitochondrial DNAs was carried out de novo with NOVOplasty 4.2 [23] using *F. musae* F31 strain as a reference and the first 715 bp from *cox1* gene as seed sequence. The obtained mitogenomes were then annotated by integrating MFannot and RNAWeasel [24]. In order to improve the final annotation, the BlastX tool was used to define the nature of the different annotated ORFs and endonucleases.

### 2.4. Alignment of Protein Genes

Mitogenomes comparison included a total of 14 concatenated sequences, which represent the 14 coding protein nucleotide sequences corresponding to the set of conserved mitochondrial genes, using the “concatenated sequences” tool on Geneious Prime Software (Biomatters Ltd., Auckland, Australia). Sequences were then aligned using the MAFFT alignment tool with default parameters and visually checked.

### 2.5. MGE Analysis (Minimap2)

Mobile genetic element analysis was performed using the distribution pattern of *F. musae* IUM_11-0508 as a reference. The goal was to identify the distribution of the same MGEs across *Fusarium* genus diversity. After MGE sequences collection, the Minimap2 tool on Geneoius Prime Software (setting a threshold of identity at 90% and minimum coverage of 75%) against all other *Fusarium* mtDNA and BlastX (coverage and identity threshold: 75 and 90% respectively) analyses were used to identify the presence of those sequences in other *Fusarium* species and other fungal species.

### 2.6. Analysis of Nad1 Intron

To confirm the sequence of the intron in nad1 in order to describe the possible path of inactivation of the nad1 endonuclease present in *F. verticillioides*, primers musaeendoF1- (5′-TGGAAAATCAGCAGGTTGACC-3′) and musaeendoR1- (5′-ACTGCTGCGTGTTCTGTCAT-3′) were designed on the coding region of the nad1 gene using primer 3 software online (https://primer3.ut.ee/, accessed on 4 November 2021). PCR amplification was carried out using Q5 master mix (NEB, Ipswich, MA, UK) in a total of 25 microliters of reaction using a PCR program that included 3 min at 95 °C, 35 cycles including 20 s at 95 °C, 20 s at 60 °C and 1 min at 72 °C, followed by a last step at 72 °C for 5 min, which was carried out in a VeritiPRO Thermal Cycler (Applied Byosystem, Waltham, MA, USA). Sequences were obtained by PCR purification and Sanger sequencing (Eurofins genomics, GER, Ebersberg, Germany). The obtained sequences were then manually checked and assembled and then aligned using the MAFFT tool in Geneious Prime Software to verify the correctness of the assembly. Moreover, BlastN, BlastX and BlastP were used to characterize the mutations occurring in the region and to identify the inactivation of endonuclease functional domains.

### 2.7. Haplotype Analysis of Mitogenomes

Whole MAFFT aligned mitogenomes using Geneious prime software were manually checked and analysed using Median Joining Network in Popart [25].

## 3. Results

### 3.1. F. musae Mitogenomes

Mitogenomes of *F. musae* strains ranged from 56,439 to 59,256 bp (Table 1). 

All mitogenomes had identical gene and tRNA distribution. Analysis carried out on the nine annotated ORFs, using the BlastP tool, showed that six of these ORFs represent mobile genetic elements (MGEs) of LAGLIDADG and GIY-YIG family, one represents a hypothetical protein, while two have to be considered ORFs with unassigned functions. 

Further investigation of *nad1*_intron revealed the presence of an inactivated endonuclease with very high similarity to the *F. verticillioides* GIY-YIG endonuclease positioned within the *nad1* gene.

**Figure 1:**
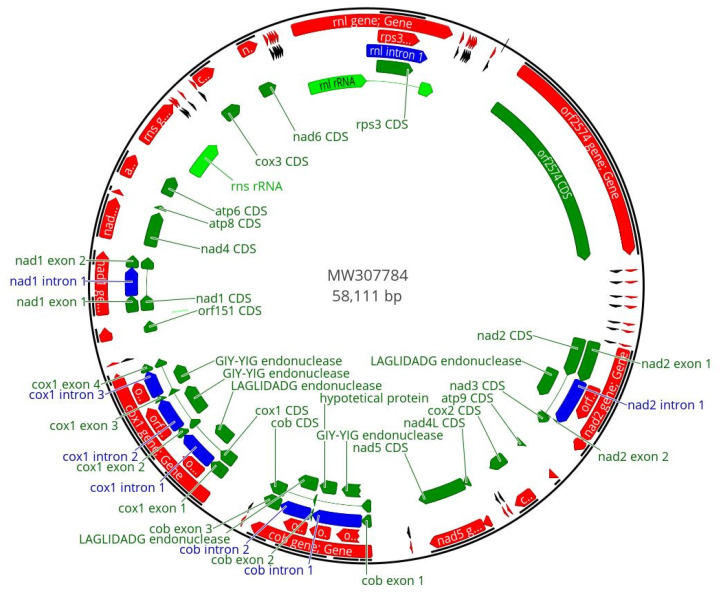
Graphical representation of circular mtDNA of *Fusarium musae* IUM_11-0508. The red colour indicates genes, the green colour refers to the coding sequences, light green refers to ribosomal subunits and the blue colour represents the introns. tRNA are represented in black.The analysis of codon usage in coding sequences did not reveal any significant difference among the whole set of analysed strains (not shown). Indeed, all the *F. musae* strains showed a high similarity for protein-coding regions. Two differences were present in strain F31: a triplet change for coding the same amino acid (Leu) and a single SNP, which causes a transversion. Strains FM_IHEM20180, FM_NRRL28993 and NRRL28997 showed one SNP with no effect on the amino acid sequence. All changes were localized in the *nad5* gene.

The final annotation of the mitogenomes of *F. musae* strains includes all the 14 protein-coding genes (*atp6*, *atp8*, *atp9*, *cob*, *cox1*, *cox2*, *cox3*, *nad1*, *nad2*, *nad3*, *nad4*, *nad4 L*, *nad5*, *nad6*); the ribosomal protein *rps3* and two ribosomal rRNA (*rns* and *rnl*); 27 tRNA genes and 2 ORFs; 6 MGEs (3 LAGLIDADG and 3 GIY-YIG positioned in *nad2* intron (*n* = 1), in *cob* introns (*n* = 2) and in *cox1* introns (*n* = 3)); 1 hypothetical protein; and 1 inactivated GIY-YIG endonuclease present in *nad1* intron (not annotated). Figure 1 shows the example of the IUM_11-0508 strain mitogenome.

**Figure 2:**
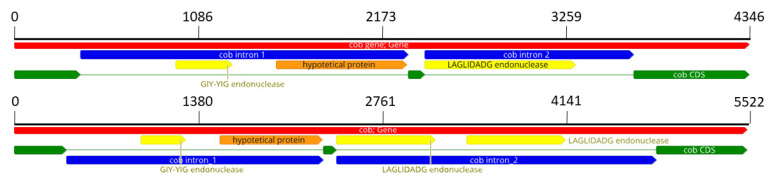
The two different patterns of the *cob* gene. On the top, the most common configuration with only one endonuclease inside the intron_2; on the bottom, the other configuration of the 4 *F. musae* strains with 2 endonucleases in intron_2. Red colour represents the gene, yellow and orange are respectively endonucleases and hypothetical protein while introns are in blue.

Size difference among mitogenomes was mostly due to variations in two regions: for strain ITEM_1149, a deletion in the ORF located between rps3 and nad2 reduces the mitogenome to 56,493, while for strains IHEM_20180, MUCL_51371, NRRL_28893 and NRRL_28897, the increased size above 59 kb is due to changes in the cob region. The *cob* gene in *F. musae* includes three exons and two introns (Figure 2). The first intron is composed of an endonuclease with GIY-YIG domain and a hypothetical protein, common in all the *F. musae* strains. The second intron shows differences within the species: in four strains (IHEM_20180, NRRL_28893, NRRL_28897, MUCL_51371), it shows a duplication of a 247 AA LAGLIDADG endonuclease, while in all the other strains, a single LAGLIDAGD of 296 AA is present (Figure 2).

BlastX analysis on these two endonucleases showed different LAGLIDADG domains for the two endonucleases (LAGLIDADG_1 and LAGLIDADG_2). Investigation for a similar pattern in other species showed that a similar duplication could be observed in *F. bactridioides*, *F. begoniae* and *F. pseudograminearum* as well as in *Cladobotryum mycophilum* (Supplementary Table S2).

*F. verticillioides* has an endonuclease within the *nad1* gene. Interestingly, *F. musae* strains also have an intron in *nad1*. Intron annotation using alignment and BlastX tool showed that *F.musae* introns have highly similar sequences to the endonuclease of *F. verticillioides*, but the presence of different mutations leading to stop codons and frame shifts suggest the inactivation of the endonuclease and the loss of functional domains. 

**Figure 3:**
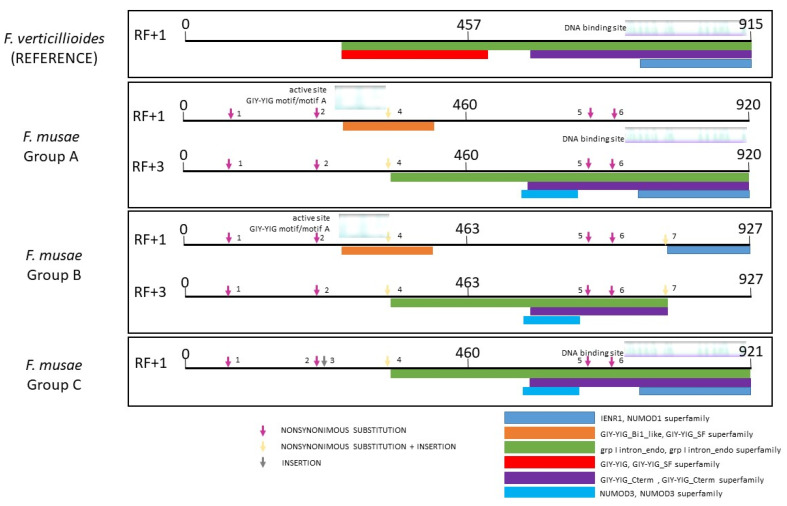
BlastX results of the intron of *nad1* gene in *F. verticillioides* and in *F. musae* strains. Types of endonuclease functional domains are visible. Numbers near arrows indicate different types of variations of the intron gene in *F. musae* strains compared to functional endonuclease in *F. verticillioides* (details on the alignment can be observed in Supplementary Figure S1). Group A includes: IHEM19981, ITEM1121, ITEM1142, ITEM1149, ITEM1250, IUM_11-0508, IUM_11-0507, MUCL51371, NRRL25673, NRRL43601, NRRL43604, NRRL43658, NRRL43682; group B: IHEM20180, NRRL25059, NRRL28893, NRRL28897; group C: F31.

Analysing *endonad1* (the most variable endonuclease within *F. musae* species) using *F. verticillioides* as a reference, we could identify three different ways of silencing the endonuclease in our *F. musae* population that are caused by insertion and nonsynonymous substitution to add stop codons in the sequence of the endonuclease (Figure 3 and Supplementary Figure S1).

### 3.2. Diversity within F. musae

Given the limited exploitation of mitogenomes for population studies, the other goal of our work was to assess whether mitochondrial diversity observed within the species could be explored to differentiate species subgroups.

**Figure 4:**
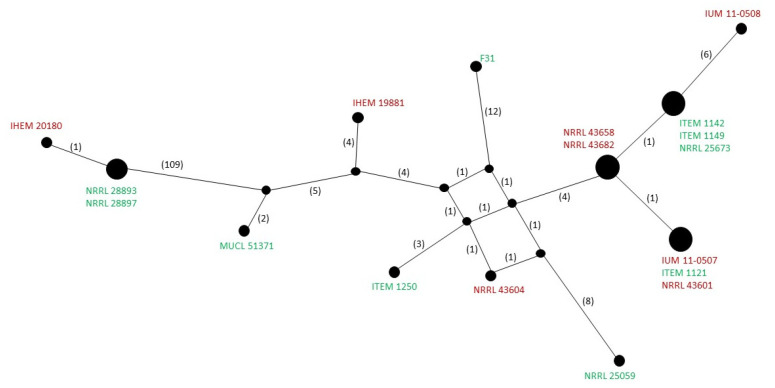
Representation of mitogenomes network haplotypes using Popart software. The red colour indicates strains isolated from human patients; the green colour indicates strains isolated from banana fruits.

Triest and Hendrickx [9] hypothesized that *F. musae* may have been transmitted from bananas to patients. To confute this hypothesis, we would expect that the hosts’ origin can be associated with different subgroups within the species, suggesting a host specialization driven by evolutionary constraints [26]. We therefore tested whether *F. musae* mitochondria diversity could be used to clearly separate strains obtained from humans and bananas. The overall diversity of mitogenome haplotypes (Figure 4) suggests that different subgroups of *F. musae* strains exist within the analysed population. 

This is consistent with the existence of different nuclear gene haplotypes [2]. Interestingly, at least one set of strains belonging to the same mitochondrial haplotype includes both human and banana derived strains from different geographic regions (ITEM_1121 from a banana fruit from Panama, IUM_11-0507 from a patient in Greece and NRRL_43601 from a patient in Maryland, USA), supporting the hypothesis that the infection of banana fruits and human patients occurs from strains with similar genetic profile. 

### 3.3. The Diagnostic Power of Mitochondrial Genomes

Another objective of the paper was to explore the use of mitochondrial diversity as a tool for the detection of species. The distribution of MEGs in a mitogenome may differentiate species. The hypothesis has been validated for other *Fusarium* species [27]. One of the major diagnostic challenges in *F. musae* is to discriminate it from *F. verticillioides*, as morphologically, the species are often misclassified [5,8].

We therefore compared the *F. musae* pattern of MGE (Supplementary Table S3) with all the other available *Fusarium* mtDNAs (Supplementary Table S1). The 6 + 1 MGEs were extracted and searched in all other mtDNAs. MGE distribution within the *Fusarium* genus showed discontinuity. In particular, *F. musae* species showed a specific pattern of MGE distribution that is unique within the *Fusarium* genus. It is possible to identify *F. musae* species based on the unique presence of two MGEs (*nad1* intron that is showing polymorphic inactivations and *endo1cox*), offering potentially a very powerful methodology to identify *F. musae*.

## 4. Discussion

This study, analysing the mitochondrial genomes of 18 *F. musae* strains, confirms previous observations based on nuclear genes (*TEF* and *RPB2*) [5,7], which showed that *F. musae* strains from banana and human patients are interspersed in the species tree. This indirectly confirms the ability of *F. musae* to cross-infect distant hosts. One strain, F31, showed a nonsynonymous substitution that may indicate some divergence within the species. Further analyses to appropriately characterize the strain are warranted. Within *F. musae* species, *nad5* proved to be the gene with higher recombinations, as observed previously in other species within the *F. fujikuroi* species complex [28]. Our mitogenome study of the *F. musae* population confirms that intergenic regions and endonucleases may be exploited to identify subgroups within a species [15]. As observed in other fungal species [29,30], we also observed that major contributors to mitogenome size diversity within a species are intron rearrangements. In our population, two regions showed variability in size: the ORF located between *rps3* and *nad2* and the intron between exon2_*cob* and exon3_*cob*. Future studies may focus on the diversity of these regions in a larger *F. musae* population. 

We observed different haplotype groups comparing whole mitochondrial diversity in *F. musae*. Previous analysis of nuclear gene haplotype diversity carried out on *F. musae* strains [2] showed the existence of nine haplotypes based on *RPB2*, *B-tub* and *TEF* diversity. Our panel of strains included representatives of six haplotypes. We could verify that nuclear gene haplotypes were all distinguished in different mitogenome groups. A larger dataset of mitogenomes needs to be analysed to verify the correspondence of nuclear gene haplotypes and mitochondrial whole diversity haplotypes. This preliminary observation seems to confirm that in *F. musae*, mitochondrial diversity is concordant with nuclear gene variations, as observed for other fungal pathogens [30], and it is therefore a valuable tool for strain typing.

The use of mitochondrial genomes for diagnostic purposes has been often proposed and explored [19,31,32,33]. We could verify that all *F. musae* strains with different geographic origins (Europe, Asia, America) and different years of isolation (from 1991 to 2013) share the same specific co-occurrence of two endonucleases that allow us to differentiate them from all other *Fusarium* species whose mitogenome is available. With the increased number of available genomes within single species, the possibility to test the hypothesis of using MGE distribution for species discrimination will be tested thoroughly.

Our study demonstrates that the variable landscape of MGEs is the most prominent type of variation among mitogenomes, including those of closely related *F. verticillioides* and *F. musae*, as shown here for the intron of *nad1*, contributing to expanding the knowledge on inter- and intraspecies mitochondrial diversity in the *F. fujikuroi* species complex [34]. Moreover, it shows that intraspecies diversity in mitochondrial size can be due to the acquisition or loss of mobile elements. The presence of strains with a human and a banana origin in the same mitochondrial haplotype group is an indirect confirmation that *F. musae* is able to cause diseases in both humans and plants. Therefore, studying the disease mechanisms of *F. musae* on hosts belonging to the two kingdoms is warranted to shed light on the infection arsenal that this cross-kingdom pathogen employs. 

## Figures and Tables

**Table 1:** List of *F. musae* strains used in this study with GeneBank accession numbers and mitogenome size and composition.

*F. musae* Strain/Feature	IUM 110508	F31	NRRL 43682	NRRL 43658	NRRL 43604	NRRL 43601	NRRL 28897	NRRL 28893	NRRL 25673	MUCL 51371	IUM 11-0507	ITEM 1250	ITEM 1149	ITEM 1142	ITEM 1121	IHEM 20180	IHEM 19881	NRRL 25059
**mtDNA**	MW 307784	MW 296866	ON 240679	ON 240982	ON 240983	ON 240980	ON 240981	ON 240987	ON 240984	ON 240985	ON 240992	ON 240991	ON 240990	ON 240989	ON 240988	ON 240986	ON 012582	MT 010916
**Length (bp)**	58,111	58,072	58,076	58,078	58,089	58,063	59,252	59,252	58,080	59,256	58,105	58,075	56,493	58,108	58,077	59,249	58,093	58,099
**Identity (%)**	REF	99.8	99.9	99.9	99.9	99.9	99.5	99.5	99.9	99.9	99.97	99.9	99.9	99.98	99.9	99.5	99.9	99.8
Protein genes *																		
**Number**	15	15	15	15	15	15	15	15	15	15	15	15	15	15	15	15	15	15
**Length (bp)**	14,148	14,148	14,148	14,148	14,148	14,148	14,148	14,148	14,148	14,148	14,148	14,148	14,148	14,148	14,148	14,148	14,148	14,148
**Identity (%)**	REF	99.96	100	100	100	100	99.99	99.99	100	100	100	100	100	100	100	99.99	100	100
**Differences**	REF	2	0	0	0	0	1	1	0	0	0	0	0	0	0	1	0	0
**Transversion**	REF	1	0	0	0	0	0	0	0	0	0	0	0	0	0	0	0	0
**MGEs**																		
Number **	6 + 1	6 + 1	6 + 1	6 + 1	6 + 1	6 + 1	7 + 1	7 + 1	6 + 1	7 + 1	6 + 1	6 + 1	6 + 1	6 + 1	6 + 1	7 + 1	6 + 1	6 + 1
**Length (bp)**	7009	7010	7009	7009	7009	7009	7464	7464	7009	7448	7009	7009	7009	7009	7009	7464	7009	7017
**Identity (%)**	REF	100%	100%	100%	100%	100%	-- ***	-- ***	100%	-- ***	100%	100%	100%	100%	100%	-- ***	100%	99.80%
**ORF**																		
**Number**	3	3	3	3	3	3	3	3	3	3	3	3	3	3	3	3	3	3
**Length (bp)**	8952	8952	8952	8952	8952	8952	8949	8949	8952	8952	8952	8955	7377	8952	8952	8949	8952	8952
**Identitcal sites**	REF	8948	8945	8945	8945	8944	8931	8931	8946	8943	8944	8945	7369	8946	8944	8931	8943	8945
**rns + tRNA**																		
**Length (bp)**	1668 + 2005	1668 + 2005	1668 + 2005	1668 + 2005	1668 + 2005	1668 + 2005	1668 + 2005	1668 + 2005	1668 + 2005	1668 + 2005	1668 + 2005	1668 + 2005	1668 + 2005	1668 + 2005	1668 + 2005	1668 + 2005	1668 + 2005	1668 + 2005
**Identity (%)**	REF	100	100	100	100	100	100	100	100	100	100	100	100	100	100	100	100	100

* Protein list: *atp6*, *atp8*, *atp9*, *cob*, *cox1*, *cox2*, *cox3*, *nad1*, *nad2*, *nad3*, *nad4*, *nad4L*, *nad5*, *nad6*, *rps3*; ** +1 represent the inactivated endonuclease; *** identity not calculated due to differences in number of endonucleases.

## Data Availability

The genome sequence data that support the findings of this study are openly available in GenBank of NCBI at [https://www.ncbi.nlm.nih.gov] (accessed on 22 May 2022)) under the accession numbers listed in Table 1. We acknowledge the fundamental contribution of mycological banks: strains used in this study are available at IHEM/MUCL (Belgium), NRRL (USA) and ITEM (Italy) and DSMZ (Germany) mycological repositories.

## Supplementary Materials

The following supporting information can be downloaded at: https://www.mdpi.com/article/10.3390/microorganisms10061115/s1, Table S1: Presence of *F. musae* endonucleases in other *Fusarium* mitogenomes; Table S2: Coverage and Identity of best hits using Blastx with endonucleases of cob gene of the four *F. musae* strains with longer mitochondrial DNA.; Table S3: Endonuclease identity in all *F. musae* strains; Figure S1: Whole nucleotide alignment of intron of *nad1* gene in *F. verticillioides* and in the 18 *F. musae* strains.
