# Supplementary material for: Exploring Mitogenomes Diversity of Fusarium musae from Banana Fruits and Human Patients
Source: Microorganisms. 2022 May 28;10(6):1115. doi: 10.3390/microorganisms10061115 (PMC9227538; doi:10.3390/microorganisms10061115)
Supplement: Supplementary file 1 [file microorganisms-10-01115-s001.zip › microorganisms-1730085-supplementary/Supplementary Figrue S1.pdf]

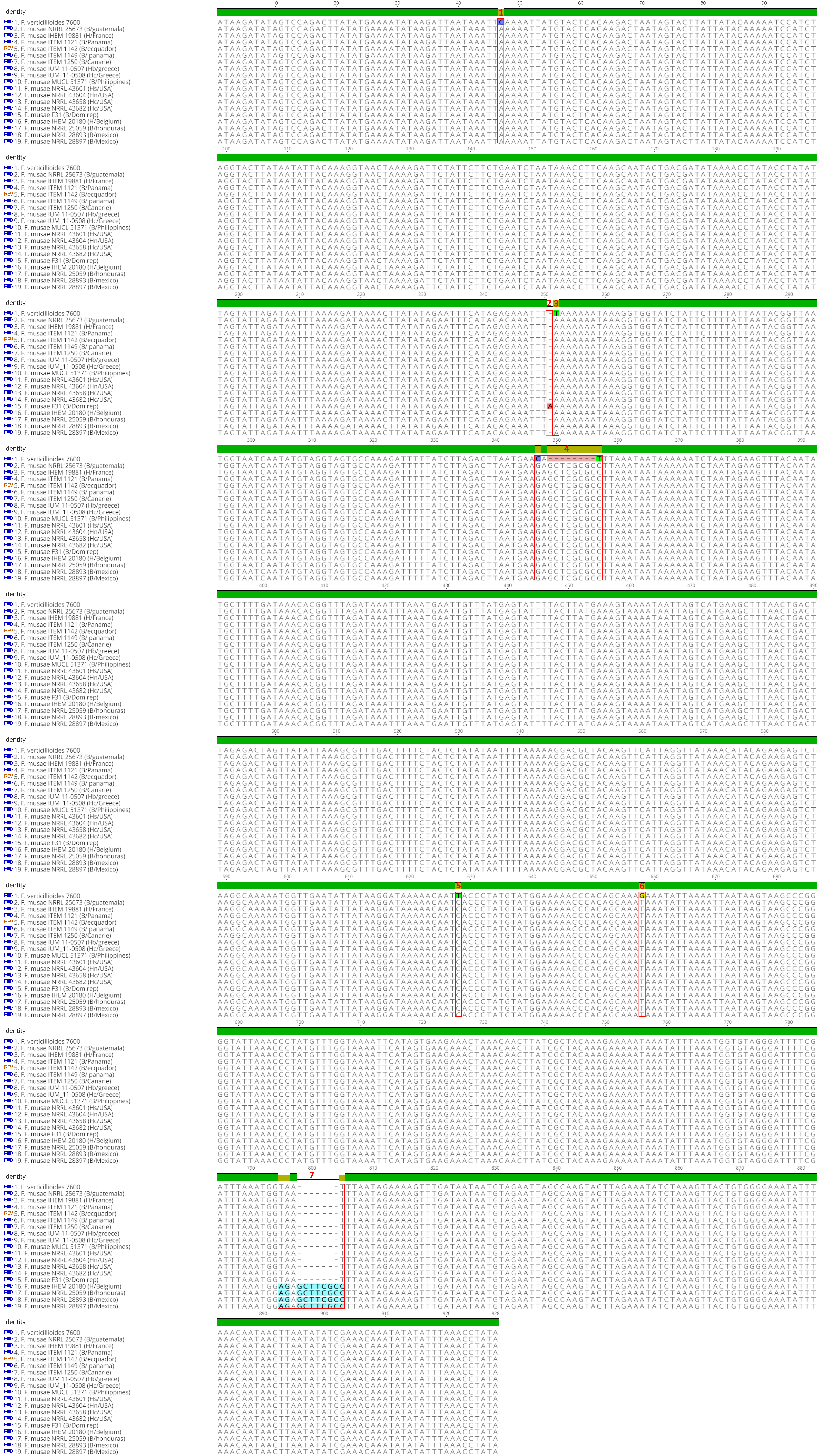
Identity
1. F. verticillioides 7600
2. F. musae NRRL 25673 (B/guatemala)
3. F. musae IHEM 19881 (H/France)
4. F. musae ITEM 1121 (B/Panama)
5. F. musae ITEM 1142 (B/ecquador)
6. F. musae ITEM 1149 (B/ panama)
7. F. musae ITEM 1250 (B/Canarie)
8. F. musae IUM 11-0507 (Hb/greece)
9. F. musae IUM_11-0508 (Hc/Greece)
10. F. musae MUCL 51371 (B/Philippines)
11. F. musae NRRL 43601 (Hs/USA)
12. F. musae NRRL 43604 (Hn/USA)
13. F. musae NRRL 43658 (Hc/USA)
14. F. musae NRRL 43682 (Hc/USA)
15. F. musae F31 (B/Dom rep)
16. F. musae IHEM 20180 (H/Belgium)
17. F. musae NRRL 25059 (B/honduras)
18. F. musae NRRL 28893 (B/mexico)
19. F. musae NRRL 28897 (B/Mexico)
FWD
REV
1
2
3
4
5
6
7
